# Improvement of hemispatial neglect by a see-through head-mounted display: a preliminary study

**DOI:** 10.1186/s12984-015-0094-5

**Published:** 2015-12-15

**Authors:** Jong Hun Kim, Byung Hwa Lee, Seok Min Go, Sang Won Seo, Kenneth M. Heilman, Duk L. Na

**Affiliations:** Department of Neurology, Dementia Center, Ilsan hospital, National Health Insurance Service, Goyang, South Korea; Department of Neurology, Samsung Medical Center, Sungkyunkwan University School of Medicine, 50 Irwon-dong, Gangnam-gu, Seoul, 135-710 South Korea; Neuroscience Center, Samsung Medical Center, Seoul, South Korea; Dr. Hwang’s Neurology clinic, Suncheon, South Korea; Department of Clinical Research Design and Evaluation, SAIHST, Sungkyunkwan University, Seoul, South Korea; Department of Neurology, University of Florida and Veterans Affairs Medical Center, Gainesville, FL USA; Department of Health Sciences and Technology, SAIHST, Sungkyunkwan University, Seoul, South Korea

**Keywords:** Hemispatial neglect, Optokinetic stimulation, Head-mounted display, Line bisection

## Abstract

**Background:**

Patients with right hemisphere damage are often unaware of, inattentive to and fail to interact with stimuli on their left side. This disorder, called hemispatial neglect, is a major source of disability. Inducing leftward ocular pursuit by optokinetic stimulation (OKS) relieves some of the signs of unilateral neglect. However, it is difficult to provide patients with a continuously moving background that is required for OKS. We studied whether OKS projected onto a see-through head-mounted display (HMD) would help treat neglect.

**Methods:**

14 patients with neglect after cerebral infarction performed line bisections on a computer screen, both with and without OKS that was either delivered by the HMD or on the same screen that was displaying the lines that were to be bisected.

**Results:**

The line bisection performances were significantly different in the four conditions (*P* < 0.001). The post hoc analyses indicated that the rightward deviation observed in the control conditions on the line bisection tasks without OKS, improved significantly with the use OKS in both the HMD and screen conditions (α < 0.05). The results between the screen and HMD conditions were also different (α < 0.05). The OKS in the HMD condition corrected patients’ rightward deviation more toward the actual midline than did the OKS provided during the screen condition.

**Conclusions:**

OKS projected onto the see-through HMD improved hemispatial neglect. The development of a portable device may aid in the treatment of neglect.

## Background

Hemispatial neglect is defined as a patient’s failure to report or respond to stimuli presented in the space opposite to a brain lesion [1]. This failure to report or respond to contralesional stimuli is often induced by inattention to stimuli located on the side of space that is contralateral to a brain lesion. The areas of the cerebral cortex that are often injured in patients with hemispatial neglect include the posterior portions of the superior and middle temporal gyri, the temporoparietal junction, the inferior parietal lobule, and the lateral prefrontal cortex [2–10]. Patients with hemispatial neglect can recover significantly during the first few months after injury. However, disabilities caused by hemispatial neglect can persist in 30 % of patients [6, 11, 12], resulting in impairments when performing activities of daily living, such as eating as well as colliding with objects situated on the contralateral side of their body, missing words when reading and even being unaware of family and friends who are situated on their left side.

Several reports have described ameliorative treatments for hemispatial neglect. Visual exploration therapy was one of the first treatments adopted for this condition [13]. Subsequently, verbal cueing, sensory stimuli, phasic alerting, and sustained attention training methods were also introduced [14, 15]. These treatments train patients to look at stimuli in their left hemispace or pay more attention to stimuli in their left hemispace [15, 16]. Various stimulation techniques such as neck vibration, caloric stimulation, cold pressor stimulation [17], prism lens, and optokinetic stimulation (OKS) have also been used to treat hemispatial neglect [18–20]. These treatments help patients to be able to shift the egocentric reference frame toward the left [11, 21]. Repetitive transcranial magnetic stimulation (TMS) on the unaffected hemisphere can also improve hemispatial neglect [22]. TMS may help improve hemispatial neglect by decreasing the transcallosal inhibition from the unaffected hemisphere [22].

There are various types of OKS, such as dots [23–25], random dot backgrounds [26], vertical strip backgrounds on a computer monitor [27], and OKS drums [21]. According to functional imaging studies, OKS activates the parieto-occipital cortex, basal ganglia, brain stem, and cerebellum [28–30]. Whereas leftward moving stimuli with OKS ameliorates left hemispatial neglect, while rightward OKS aggravates left hemispatial neglect [21, 31]. OKS is one of the most effective treatments for hemispatial neglect [11], and is easy to administer. In addition, OKS can affect other symptoms of neglect syndrome including even helping patients better attend to contralateral stimuli that are not visual, such as auditory stimuli [32]. OKS can also help improve distorted body orientation, motor neglect and tactile extinction [24, 25, 31, 33].

There may be two shortcomings of OKS when it is applied to patients with hemispatial neglect. First, whether the effects of treatment with OKS can even last for some time after OKS is discontinued is controversial, although two recent randomized control trials showed that the treatment effect of OKS persisted with hemispatial neglect received repetitive OKS for treatment (one with 5 sessions about 50 min [34], the other with 20 sessions about 30 min [35]). Second, the treatment using OKS outside the laboratory has been impractical because when patients are viewing objects in their environment, the OKS should take place in the background in order for the OKS to be effective. More specifically, providing OKS in the background during the times a person is performing their activities of daily living as well as instrumental activities cannot be practically performed. In order to overcome these shortcomings, instead of providing OKS in the background, we wanted to learn if providing OKS in the foreground may help reduce patients’ hemispatial neglect. Thus, we wanted to investigate if OKS projected onto a see-through head-mounted display (HMD) would help treat hemispatial neglect, as well as examining if this treatment was as effective as using background OKS. If HMD with OKS reduces the signs of neglect, it could then be used as an effective treatment.

## Methods

### Participants

We recruited patients who had been admitted to the Department of Neurology of the Samsung Medical Center from October 2007 to September 2009. Among the patients with lesions of the right hemisphere, we selected 14 patients (5 woman, mean age, 73.1 ± 5.8 years) with evidence of hemispatial neglect. The presence of hemispatial neglect was determined by the criteria described in the following section. This study was approved by the Institutional Review Board of the Samsung Medical Center, Seoul, South Korea and we obtained written consent for participation in this study from the participants or their next-of-kin.

### Selection of patients with hemispatial neglect

In order to screen for the presence of hemispatial neglect, a line bisection test was performed. A solid line (length, 242 mm; thickness, 1.5 mm) was placed at the center of 297 by 210 mm sheet of white paper. Our previous study involving 80 normal individuals yielded a mean bisection deviation of −1.0 ± 3.5 mm (negative sign indicates a leftward deviation). In these normal participants’ performance of line bisections, 2 standard deviations (SD) ranged from −8.0 mm to 6.0 mm [36]. Therefore, we regarded patients as having left hemispatial neglect when they deviated on their 10 attempted line bisections an average of 6.0 mm or greater.

### Experimental design

**Fig. 1:**
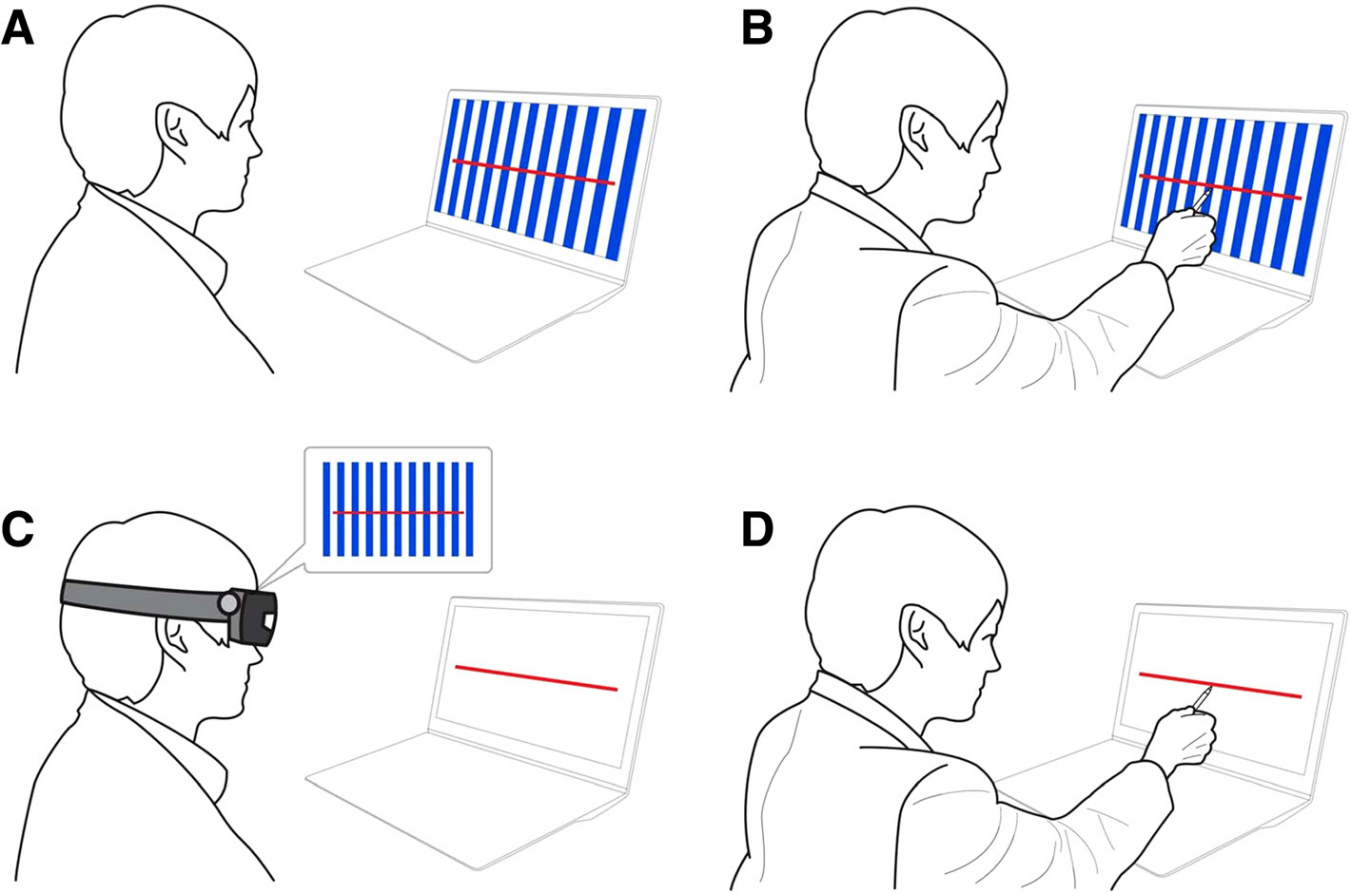
Experimental designs. **a** Screen condition: the optokinetic stimulation (OKS) consists of white and blue vertical stripes that are either stationary or moving leftward. **b** Patients are requested to point to the subjective midpoint on the red line on the LCD screen with a black pen in his right hand and a researcher clicked the point with mouse on the screen. **c** HMD condition: the patients are able to see the horizontal red line on the screen through the HMD while the OKS appears on the HMD. **d** Patients perform line bisection as illustrated in the screen condition (**b**)

The patients performed line bisection tasks in four different conditions (Fig. 1). In one condition (the screen condition without OKS) the line was observed on a computer screen. There was no OKS presented on the screen (vertical stripes were present on the screen but was stationary) and the participants did not use the HMD. In a second screen condition the line was presented with OKS in the background but no HMD was used. There were also two HMD conditions, without/with OKS (Fig. 1). Herein, we refer to these four conditions as screen –OKS, screen + OKS, HMD –OKS, and HMD + OKS. We used amiraglos SX® (Deocom, Seoul, Korea) for the see-through HMD. In the screen condition, the OKS and red horizontal line appeared on a liquid crystal display (LCD) screen (Fig. 1a, b), and patients executed line bisection without wearing an HMD. The size of the LCD screen was 15 in. (304 × 228 mm). The red line (length, 262 mm; thickness, 2 mm; width, 740 pixels) was located in the center of the screen. The OKS consisted of white and blue vertical stripes with a width of 140 mm. During the + OKS trial these stripes moved leftward at a velocity of 8.9 cm/s (10.1 °/s). In the HMD condition, the OKS appeared on the HMD and the patients were able to see the horizontal red line on the screen through the HMD (Fig. 1c, d). There were 6 line bisection trials in each 4 conditions, and therefore, each patient underwent 24 trials (six trials × four conditions). The first seven patients were tested with the screen conditions first and then tested in the HMD condition. This order of the conditions was reversed for the last seven participants. Within each screen or HMD condition, the order of OKS movements (static versus leftward moving) was randomly arranged. The horizontal distance between the lines on the screen and the subjects’ eyes were 50 cm. We aligned the midline of the monitor with the midsagittal plane of the subject’s head and body. In each trial, patients were allowed to finish the bisection task with no time limits. Since most patients were unfamiliar with using computer mouse, patients were requested to point to the subjective midpoint on the red line on the LCD screen with a black pen in their right hand and a researcher clicked this point with mouse on the screen. Then, the computer program automatically stored the location of the point. Performance on these line bisection tests were measured by the degree of pixel deviation. The OKS and test management software was made with Visual C++ (version 6.0; Microsoft, Redmond, WA) and Direct X (version 6.0; Microsoft, Redmond, WA).

### Statistical analyses

Our experiment model fit well to a completely randomized design with subsampling. In other words, we can regard the four different conditions as four treatments, and the six repetitions on each condition as six subsamples. Therefore, the result was analyzed using analysis of variance (ANOVA) of a randomized complete block design with subsampling. The post hoc analyses were performed using Tukey’s test. All the data were analyzed using SAS (version 9.2; SAS Institute, Cary, NC).

## Results

**Fig. 2:**
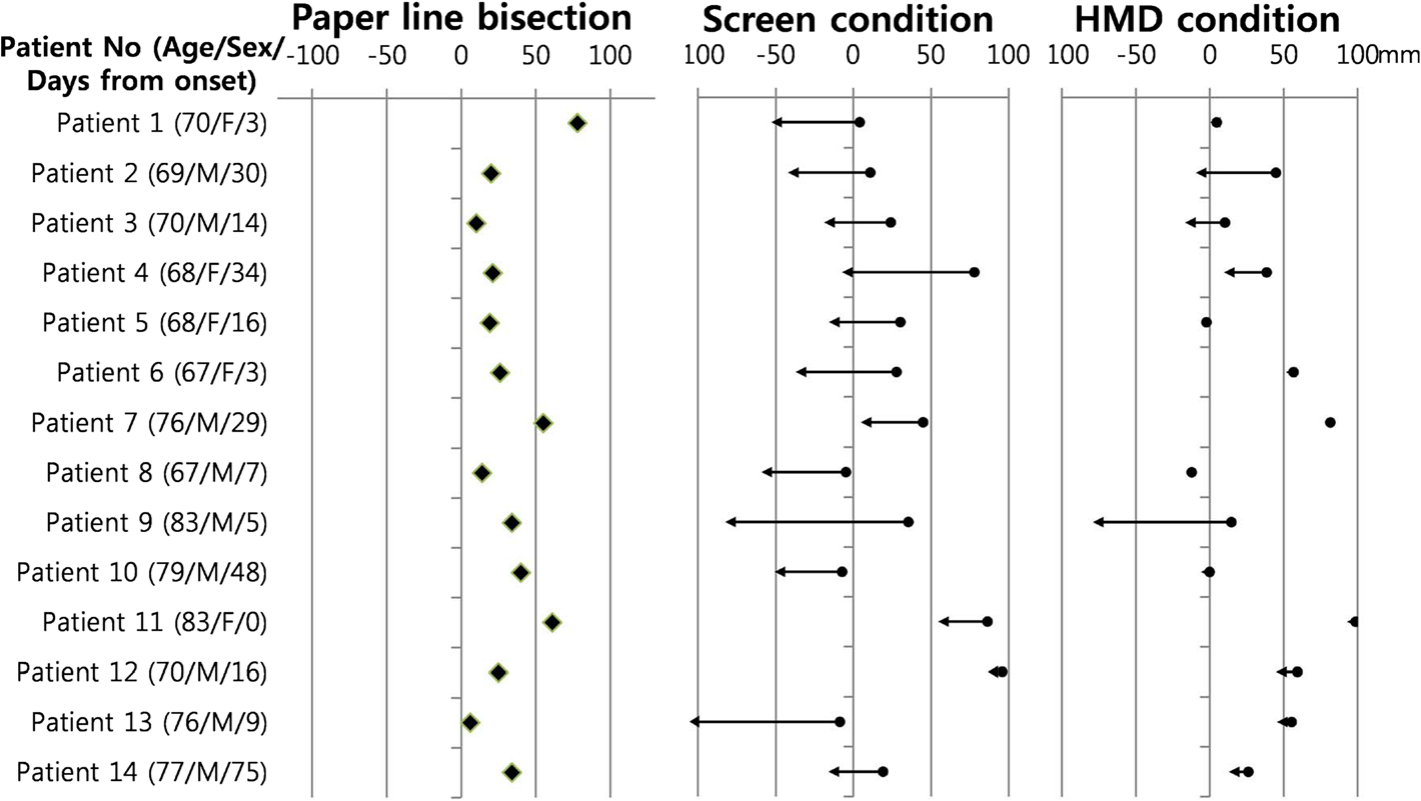
An illustration of bisection performances in all patients with hemispatial neglect (*N* = 14). In the line bisection tests on paper at screening (leftmost column), diamonds represent mean values of ten trials of line bisection. In the screen (middle column) and HMD (rightmost column) conditions, the closed circles are the mean positions of line bisection tests in OKS– and the arrow heads are mean positions of line bisection tests in OKS+. Refer to the methods section for details

**Fig. 3:**
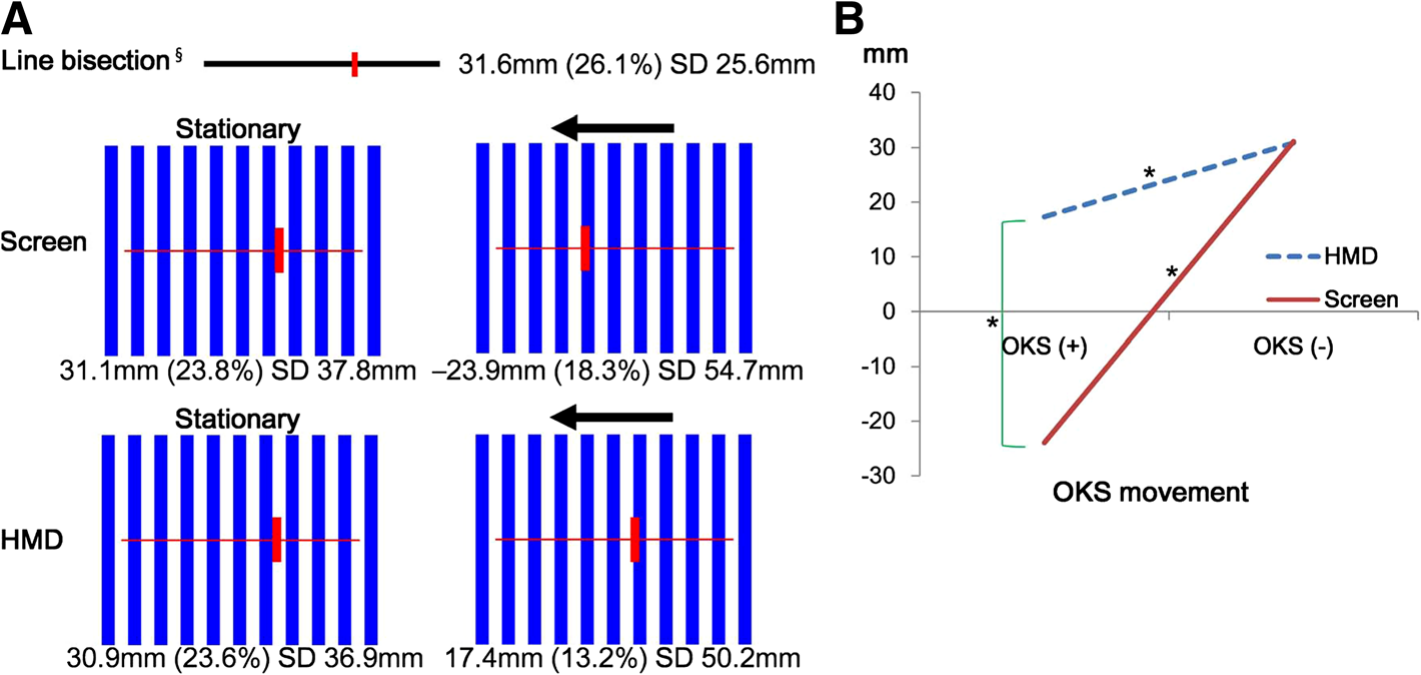
A schematic overall results (**a**) ‘Stationary’ is defined by no movement of the blue strips (OKS–). The leftward arrow indicates leftward movement of the blue strips (OKS+). **b** There were statistically significant differences (marked with *) between conditions with and without OKS in both screen and HMD conditions according to Tukey’s test after ANOVA of a randomized complete block design with subsampling. Plus numbers mean rightward deviation in the line bisection tasks and vice versa. ^§^ The results of line bisection in the screening test done on the paper. Abbreviations: OKS+, leftward OKS movement; OKS–, no OKS movement; HMD, head-mounted display; SD, standard deviation

We tested the effects of screen + OKS, screen –OKS, HMD + OKS and HMD –OKS on the line bisection tasks. The deviations from the midline are expressed as millimeters translated from pixel dimensions. Leftward deviation was coded as minus value, whereas rightward deviation as plus value. The bisection performances of each patient are presented in Fig. 2, and summarized results of the four conditions (screen –OKS, screen + OKS, HMD –OKS, and HMD + OKS) are shown in Fig. 3 and Table 1. There were statistically significant differences among these four conditions (*P* < 0.001). First, the results of screen –OKS (31.1 ± 37.8 mm) and HMD –OKS (30.9 ± 36.9 mm) conditions did not differ from each other. Second, the results of screen + OKS (−23.9 ± 54.7 mm) condition were significantly leftward compared to those of the screen –OKS (31.1 ± 37.8 mm) condition. Third, the results of HMD + OKS (17.4 ± 50.2 mm) condition were also significantly leftward compared to those of HMD –OKS (30.9 ± 36.9 mm) condition. Finally, the degree of the effect of OKS was different between the HMD and screen conditions: the OKS projected onto the screen overcorrected hemispatial neglect with leftward deviation.

**Table 1:** Results of the experiments

	DF	SS	MS	F	*P*
Conditions	3	170229.6	56743.2	115.8	<0.001
Patient	13	375359.5	28873.8		
Experimental error	39	176436.4	4524.0		
Observational error	280	137237.7	490.1		
Total	335	859263.2			

We performed statistical analyses using ANOVA of a randomized complete block design with subsampling

*Abbreviations*: *DF* degree of freedom, *SS* sum of squares, *MS*, mean of squares

## Discussion

The results of the screen condition replicated previous findings that OKS has a beneficial effect on hemispatial neglect. The results of the see-through MHD condition, however, may be the first to demonstrate that see-through HMD may be applicable to the treatment of hemispatial neglect. Although the extent of the correction created by the OKS projected onto screen was larger than that created by the OKS HMD, the latter did decrease the patients’ rightward deviation, whereas the former overcorrected the hemispatial neglect (Fig. 3b) such that these patients were now neglecting right hemispace.

Our results suggest that OKS projected onto the see-through HMD can be a potential treatment for hemispatial neglect. Numerous studies suggested that OKS can ameliorate hemispatial neglect [20, 31, 37]. However, OKS has not been used to help reduce the disability associated with spatial neglect because the OKS cannot be overlapped with real objects. The see-through HMD device, however, allows patients to see scenes or objects in real life and at the same time to project OKS.

When viewing objects and scenes, seeing moving stimuli may be either distracting, uncomfortable or even annoying. A prior study showed that administration of OKS, even in peripheral vision can also be effective in the treatment of hemispatial neglect. [11] Therefore, the HMD can possibly be altered to make patients more comfortable by applying OKS only to the periphery of the visual field so that patients can see objects in the central part of visual field more clearly and comfortably. In addition, the frequent use OKS over weeks or even months may also allow the brain to reorganize with reduction of the signs and symptoms of neglect even when this treatment is discontinued and thus future studies will have to test this possible treatment.

In the screen condition, leftward OKS overcorrected left hemispatial neglect and the patients who had a rightward deviation exhibited a deviation to the left side. This finding suggests that patients with impairments in the allocation of spatial inattention may be more distracted by background movement than are normal subjects [37], and such over-distractibility by leftward OKS may limit its clinical utilization. However, further research will have to investigate whether such overcorrection may have a stronger after-effect and whether the extent of overcorrection can be lessened by such factors as slower velocity of the OKS and reduced number of moving bars.

In this study, the magnitude of leftward correction in the HMD + OKS condition was less than that of the screen + OKS condition. Thus, when compared to the screen + OKS condition the leftward HMD + OKS condition distracted patients less and better corrected the patients’ rightward deviation so that it was near to the midline. One possible reason for this difference is that in the screen condition, the patients might have been able to focus on the line and OKS at the same time. In contrast, in the HMD condition, the patients might have focused more on the line than the OKS, resulting in less distraction by + OKS in the HMD condition. Thus in the HMD + OKS condition, patients might be able to perform daily tasks with less distraction then when + OKS and objects are presented on a screen.

The present study had several limitations. First, we tested the effect of OKS only on the line bisection task. There are many other tasks that are impaired in patients with hemispatial neglect such as cancellation, drawing, and even visual imagery. It has been reported that some patients who show severe defects on cancellation tasks perform normally on line bisection tasks, and vice versa [38, 39] and future studies will have to assess the effects of HMD + OKS treatment on these other signs of spatial neglect. Second, we did not test whether the see-through HMD was able to improve patients’ activities of daily living and the performance of instrumental activities. Lastly, we have not investigated whether the effect of OKS in the HMD is sustained across time with continued stimulation and the interaction of OKS with other interventions that do not involve visual feedback such as pharmacological interventions [40]. Future research should be directed toward assessing these important issues.

## Conclusion

Our study showed that the OKS delivered by a see-through HMD can be a potential treatment strategy for hemispatial neglect. Further studies for implanting our strategy into advanced devices are required.

## Abbreviations

OKS: Optokinetic stimulation
HMD: Head-mounted display
TMS: Transcranial magnetic stimulation
LCD: Liquid crystal display
ANOVA: Analysis of variance
AD: Alzheimer’s disease
